# Genome assembly and annotation of the mermithid nematode *Mermis nigrescens*

**DOI:** 10.1093/g3journal/jkae023

**Published:** 2024-02-01

**Authors:** Upendra R Bhattarai, Robert Poulin, Neil J Gemmell, Eddy Dowle

**Affiliations:** Department of Anatomy, University of Otago, Dunedin 9016, New Zealand; Department of Organismic & Evolutionary Biology, Harvard University, Cambridge, MA 02138, USA; Department of Zoology, University of Otago, Dunedin 9016, New Zealand; Department of Anatomy, University of Otago, Dunedin 9016, New Zealand; Department of Anatomy, University of Otago, Dunedin 9016, New Zealand

**Keywords:** *Mermis nigrescens*, nematode, hybrid genome assembly, repeatome, genome annotation

## Abstract

Genetic studies of nematodes have been dominated by *Caenorhabditis elegans* as a model species. A lack of genomic resources has limited the expansion of genetic research to other groups of nematodes. Here, we report a draft genome assembly of a mermithid nematode, *Mermis nigrescens.* Mermithidae are insect parasitic nematodes with hosts including a wide range of terrestrial arthropods. We sequenced, assembled, and annotated the whole genome of *M. nigrescens* using nanopore long reads and 10X Chromium link reads. The assembly is 524 Mb in size consisting of 867 scaffolds. The N50 value is 2.42 Mb, and half of the assembly is in the 30 longest scaffolds. The assembly BUSCO score from the eukaryotic database (eukaryota_odb10) indicates that the genome is 86.7% complete and 5.1% partial. The genome has a high level of heterozygosity (6.6%) with a repeat content of 83.98%. mRNA-seq reads from different sized nematodes (≤2 cm, 3.5–7 cm, and >7 cm body length) representing different developmental stages were also generated and used for the genome annotation. Using *ab initio* and evidence-based gene model predictions, 12,313 protein-coding genes and 24,186 mRNAs were annotated. These genomic resources will help researchers investigate the various aspects of the biology and host–parasite interactions of mermithid nematodes.

## Introduction

Nematodes are a highly diverse group with over 1 million estimated species (Scheffers *et al.* 2012; Smythe *et al.* 2019). They are ubiquitous, occurring in habitats ranging from deep oceans to mountain peaks (Schratzberger *et al.* 2019). Most animals and plants species are host to at least one species of parasitic nematode (Blaxter and Koutsovoulos 2015). Despite representing an important component of all natural ecosystems (Ferris 2010; Cardoso *et al*. 2016), most nematode species remain undescribed (Dobson *et al.* 2008).

Nematodes fall into two broad classes based on both molecular and morphological systematics: Chromadorea and Enoplea (Blaxter *et al.* 1998; Liu *et al.* 2013). They have a conserved body plan with similar structural organization and cellular morphologies (Basyoni and Rizk 2016). However, comparative analyses have highlighted the molecular and physiological diversity within the phylum (Mitreva *et al.* 2005; International Helminth Genomes Consortium 1975). *Caenorhabditis elegans* is a Chromadorean that has been at the forefront of genetic research as a model species. In contrast, there exists very little genomic information from the members of class Enoplea. Most studies on Enoplea focus on *Trichinella spiralis* (Enoplea: Trichocephalida) because of its importance as a mammalian parasite (Mitreva *et al.* 2011). Mermithida represents an order within Enoplea where most members are endoparasites of arthropods. However, among Mermithida, only the genome of *Romanomermis culicivorax* (Dorylaimia: Mermithidae) is publicly available (Schiffer *et al.* 2013).

Mermithids are obligate endoparasitic nematodes. They represent a large portion of the understudied nematode class Enoplea. There are over 100 described mermithid species; however, their biology and genetics are scarcely studied (International Helminth Genomes Consortium 1975; Presswell *et al.* 2015). Mermithids are parasitic in different kinds of invertebrates like insects, spiders, leeches, and crustaceans. However, insects are by far the most common hosts, ∼15 different orders of insects are host to different mermithids (Nickle 1972). These nematodes lack a functional gut and absorb nutrients through their outer cuticle from the insect's hemolymph and store them in their trophosome. The free-living form will rely on those stored reserves, so they depend heavily on their host for their nutritional requirements (Petersen 1985). They are relatively large nematodes with a wide distribution spanning the Americas, Eurasia, Australia, and New Zealand. *Mermis subnigrescens* was reported from New Zealand as early as 1989 by Thomas (cited by Presswell *et al*. 2015). Later *M. subnigrescens* was synonymized with *M. nigrescens* (Nickle 1972).

Mermithids are reported to be facultatively parthenogenetic (Christie 1937). Environmental signaling is thought to play a primary role in their sex determination (Christie 1929). The first individual establishing in a host usually develops as female, with the sex of individuals arriving later determined in response to others in the pool. However, the genetic mechanisms of sex determination for mermithid individuals in a population are unknown.

Several mermithids manipulate their host's behavior for their own benefit (Herbison *et al.* 2019). Adult mermithids are free-living; after completion of their development within an arthropod host, they must emerge from the host to mate and lay eggs. As they are prone to desiccation, they must emerge in water. Since many mermithids parasitize terrestrial arthropod hosts, the need to emerge from the host in water has selected in several mermithids the ability of inducing water-seeking behavior in their host. For example, the mermithid nematode *Strelkovimermis spiculatus* modifies the behavior of its adult female mosquito host to make it seek water instead of a blood meal, providing a dispersal means for the nematodes and ensuring their return to a suitable habitat for reproduction (Allahverdipour *et al.* 2019). Similarly, *Mermis nigrescens* induces water-seeking behavior in its terrestrial arthropod host, enabling the nematodes to emerge into a favorable environment to continue their life cycle (Presswell *et al.* 2015; Herbison *et al.* 2019).

Here, we present a high-quality genome assembly of *M. nigrescens*. The life cycle, morphological, and molecular characteristics of *M. nigrescens* have been well studied (Baker and Capinera 1997; Presswell *et al.* 2015). However, the lack of whole-genome information greatly limits the genetic investigations of its biology, behavior, and adaptations. This genome will also fill the knowledge and resource gap for Enoplean genomes. It will further facilitate the application of a range of molecular tools and approaches to study the genetic underpinnings of successful host exploitation by mermithids.

## Materials and methods

### Sampling and nucleotide extractions

Nematodes (*M. nigrescens*) were dissected out of European earwigs (*Forficula auricularia*) collected from the Dunedin Botanic Garden (latitude: −45° 51′ 27.59″ S; longitude: 170° 31′ 15.56″ E) and reared in a temperature-controlled room (temperature: cycling from 15 to 12°C, day/night; photoperiod of L:D 16:8) in the Department of Zoology, University of Otago, Dunedin. Nematodes thus obtained were snap-frozen in liquid nitrogen and stored at −80°C until further use. Individual nematodes were used for each nucleotide extraction. Individuals were not sexed prior to sequencing due to the difficulty in achieving this in a manner timely enough to maintain high-quality RNA and DNA samples. DNA extracted using DNeasy Blood & Tissue Kit (Qiagen, USA) was used for nanopore sequencing. DNA extracted using Nanobind Tissue Big DNA kit (Circulomics, USA) was used for 10x Chromium linked-read library preparation. RNase treatment to remove RNA was performed using 4μl of RNase A (10 mg/ml) per 200μl of template following DNA extraction. DNA samples were quantified, and quality assessed using a Qubit 2.0 Fluorometer (Life Technologies, USA) and Nanodrop. High-quality DNA samples were stored at −20°C and were used within a week of extraction.

Total RNA from different sized nematodes (≤2 cm, 3.5–7 cm, and >7 cm body length) representing different developmental stages was extracted using Direct-zol RNA MicroPrep kit (Zymo Research) following the manufacturer's instruction and stored at −80°C until further use. RNA from 5 individuals from each size category (small, medium, and large) were individually extracted and later pooled at equimolar concentration to make a single sequencing library for each category. Samples were quantified and quality checked on a Qubit 2.0 Fluorometer (Life Technologies, USA) and Nanodrop. RNA integrity for the pooled samples was assessed using a Fragment Analyzer (Advanced Analytical Technologies Inc., USA) at the Otago Genomics Facility (OGF), University of Otago, Dunedin, New Zealand. The RNA quality number values ranged from 8.3 to 9.5, indicating high-quality samples.

### Library preparation and sequencing

A long-read sequencing library for Oxford minion was prepared with 416.5 ng of genomic DNA using a ligation sequencing kit (SQK-LSK109) (Oxford Nanopore Technologies, Oxford, UK) following the manufacturer's instructions. Lambda phage (DNA CS) was used as a positive control. The prepared library was sequenced with R9 chemistry MinION flow cell (FLO-MIN106) (Oxford Nanopore Technologies, UK).

A linked-read library was prepared at the Genetic Analysis Services (GAS), University of Otago (Dunedin, New Zealand). DNA was size selected for fragments over 40 kbp using a Blue Pippin (Sage Science, USA). A 10x Chromium linked-read (10x Genomics, USA) library was prepared following the manufacturer's instructions. The library was sequenced on the Illumina Nova-seq platform to generate paired-end reads (2 × 151 bp) at the Garvan Institute, Australia.

TruSeq stranded mRNA libraries were prepared and sequenced at OGF as 2 × 100 bp paired-end reads across 2 Rapid V2 flowcell lanes of HiSeq 2500.

### Genome size estimation

Genome size was estimated with flow cytometry analysis at Flowjoanna (Palmerston North, New Zealand). Two whole worm samples were prepared following a protocol described in our earlier study (Bhattarai *et al.* 2022). Rooster red blood cells from the domestic chicken (*Gallus gallus*) were used a reference. Samples were then processed on a FACSCalibur (BD Biosciences, USA) and the data were analyzed using Flowjo (BD Biosciences, USA).

### Bioinformatic pipeline

**Fig. 1.**
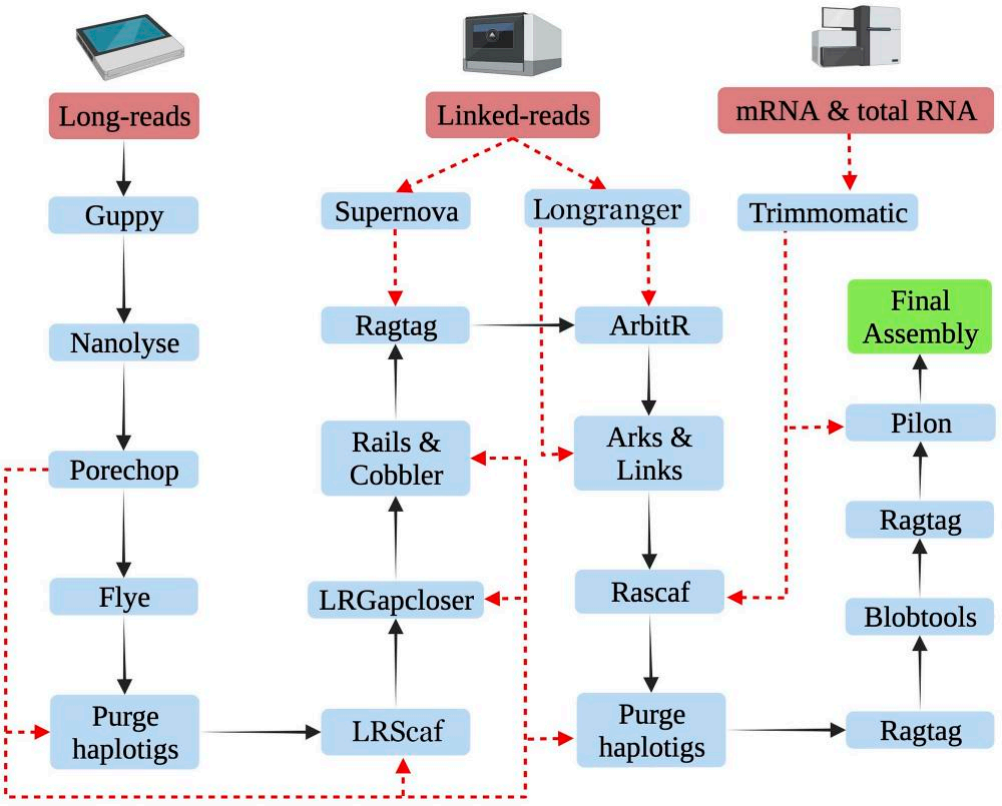
Schematic representation of the assembly pipeline for the *Mermis nigrescens* genome. The black arrows represent the workflow, and the red dotted lines represent the input data in the pipeline (created with BioRender.com).

We used various software and packages to assemble and refine the genome (Fig. 1). The pipeline and the scripts used in this project are available on GitHub (https://github.com/upendrabhattarai/Nematode_Genome_Project). The bioinformatics software and packages were run in New Zealand eScience Infrastructure (NeSI).

### Genome and transcriptome assembly

All the Nanopore reads were basecalled using Guppy (v.5.0.7) post-sequencing. Quality control of the long-read data involved Nanolyse (v1.2.0) (De Coster *et al.* 2018) and Porechop (v.0.2.4) (Wick *et al.* 2017). Reads were assembled using Flye (v.2.9) assembler (Kolmogorov *et al.* 2019) with default parameters. The paired-end reads from the chromium library were assembled using supernova (Weisenfeld *et al.* 2017). BUSCO (v.4.1.4) (Simão *et al.* 2015) with eukaryota_odb10 and nematoda_odb10 databases and Quast (v.5.0.2) (Gurevich *et al.* 2013) were used to evaluate the assembly statistics. The assembly from long reads outperformed the linked reads for the BUSCO completeness and assembly contiguity. Therefore, long-read assembly was used as a primary assembly, and the linked-read assembly was used for scaffolding later in the pipeline (Fig. 1). Multiple scaffolding and gap-closing steps were performed using Lrscaf (v.1.1.11) (Qin *et al.* 2019), Rails (v.1.5.1) and Cobbler (v.0.6.1) (Warren 2016), Ragtag (v.2.1.0) (Alonge *et al.* 2019), ArbitR (v.0.2) (Hiltunen *et al.* 2021), Arks (v.1.0.4) (Coombe *et al.* 2018), Links (v.1.8.7) (Warren *et al.* 2015), and Lrgapcloser (Xu *et al.* 2019). Linked reads were mapped to the assembly with Longranger (v.2.2.2) (Marks *et al.* 2019) prior to scaffolding. mRNA-seq reads obtained for genome annotation purposes, and total RNA-seq reads sequenced for another project (manuscript under preparation) were also used for scaffolding the assembly with Rascaf (Song *et al.* 2016). Purgehaplotigs (Roach *et al.* 2018) removed haplotigs from the assembly. Removed haplotig sequences were used to further scaffold the assembly with Ragtag (v.2.1.0). Blobtools2 (v.2.6.4) (Laetsch and Blaxter 2017) was used to remove and discard bacterial reads and remove low coverage (<5× coverage) and short (<1,000 bp) scaffolds. Low coverage and short scaffolds were then used to scaffold the filtered assembly with Ragtag (v.2.1.0) (Alonge *et al.* 2019). Finally, Pilon (v.1.24) was used to polish the assembly using mRNA-seq data.

The adapters and low-quality reads from mRNA-seq data were filtered using Trimmomatic (v.0.38) (Bolger *et al.* 2014) with options HEADCROP:15 LEADING:3 TRAILING:3 SLIDINGWINDOW:4:20 MINLEN:35. Clean data were de novo assembled with Trinity (v.2.13.2) (Grabherr *et al.* 2011) using all the default parameters.

### Repetitive content masking

A custom repeat library was generated using *de novo* and homology-based identifiers, including LTRharvest (Ellinghaus *et al.* 2008), LTRdigest (Steinbiss *et al.* 2009), RepeatModeler (Flynn *et al.* 2020), TransposonPSI (Brian Haas 2010), SINEBase (Vassetzky and Kramerov 2013), and MITE-Tracker (Crescente *et al.* 2018). Individual libraries from each software were concatenated, and sequences with more than 80% similarity were merged to remove redundancy using usearch (v.11.0.667) (Edgar 2010). The library was then classified using RepeatClassifier. Sequences with unknown categories in the library were mapped against the reviewed nematode database from UniProtKB/Swiss-Prot database (*e* < 1e^−01^); if not annotated as repeat sequences, they were removed from the library. The final repeat library was used in RepeatMasker (v.4.1.0) (Chen 2004) to mask the repeats and generate a report. The repeat library was used as an input to the MAKER2 pipeline (Holt and Yandell 2011). Tandem repeats were also identified using the TRF (v. 4.09.1) pipeline (Benson 1999).

### Genome annotation

The assembly was annotated using evidence-based and *ab initio* gene model predictions through MAKER2 (v.2.31.9) (Holt and Yandell 2011) and Braker (v.2.16) (Hoff *et al.* 2019) pipelines. The first round of MAKER2 was run with 180,630 mRNA transcripts *de novo* assembled with the Trinity (v.2.13.2) pipeline (Grabherr *et al.* 2011) along with 511,117 mRNA and 1,148,233 protein sequences from all the Dorylaimia species available in NCBI and WormBase (https://wormbase.org) databases. mRNA-seq reads and output from GenMark-ES were used to train Augustus with the Braker pipeline. SNAP was trained after each round of MAKER2. Trained SNAP and Augustus were used for 2 more iterations of the MAKER2 pipeline.

We used InterProScan (v.5.51–85.0) (Jones *et al.* 2014) for the functional annotation on the predicted protein sequences from MAKER and retrieved InterPro ID, PFAM domains, and Gene Ontology (GO) terms. Furthermore, the Uniprot database with BLAST was used to assign gene descriptors to each transcript based on the best hit.

## Results and discussion

### Genome size estimates

The flow cytometry estimate of the nematode genome size is 814.185 ± 24.2 Mb (mean ± SD). However, the nematode samples yielded very low nuclei counts, i.e. as low as <200 total nuclei for 1 of the samples. Given that this is a whole animal digest, and the very low nuclei counts in the samples, the calculated pg/nucleus may not be accurate. To get better genome size estimates by flow cytometry in this nematode, we recommend increasing the sample size and replicated measurements from the nuclei suspension in future experiments.

### Genome and transcriptome assembly

A total of 6.9 Gb of sequencing data was generated from a Nanopore minion flowcell run consisting of 530,888 reads with an N50 length of 17,849 bp (Supplementary Table 1). Flye (v.2.7.1) produced the primary long-read assembly with 16,414 contigs with N50 of 94,974 bp and a total length of ∼714 Mb. Quast reported 88.45% complete BUSCO from the Eukaryote database for this assembly (Table 1).

**Table 1.** Statistics for the genome assembly of *Mermis nigrescens*.

	Assembly length	No. of scaffolds	N50	L50	Ns per 100 kbp	BUSCO% Quast (eukaryota_odb10)	
						Complete	Partial
Flye	714,265,163	16,414	94,974	2,079	0.45	88.45	4.29
Supernova	582,450,576	147,205	9,664	12,318	732.59	20.13	21.45
Final assembly	524,220,005	867	2,429,002	30	240.96	86.70	5.10

The 10x Chromium library yielded 450.3 million paired-end reads. The Supernova assembler produced an assembly of 582.5 Mb with 147,205 scaffolds and N50 of 9,664 bp. Quast reported only 20.13% of complete BUSCO genes (Table 1). Comparing the contiguity and completeness of the long-read vs linked-read assemblies, we used long-read assembly as the primary assembly and the linked-read assembly was used later for scaffolding (Fig. 1).

**Fig. 2.**
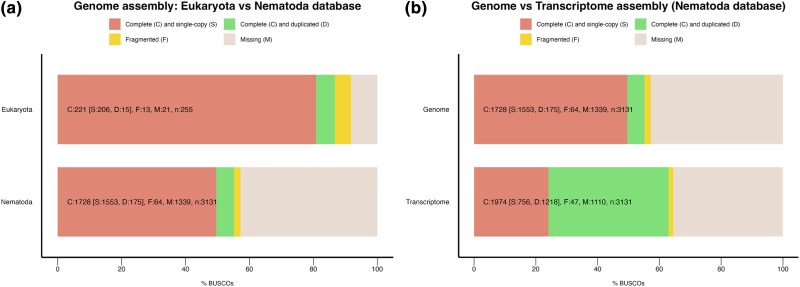
The BUSCO (v.4.1.4) analysis of the genome and transcriptome assembly of *Mermis nigrescens*. a) Comparison between the Eukaryote (eukaryota_odb10) and the Nematode (nematoda_odb10) database for BUSCO scores for the assembled genome. b) Comparison between genome and transcriptome assemblies for BUSCO scores with the nematoda_odb10 database.

The assembly statistics after each round of processing are presented in Supplementary Table 2. The final assembly has a size of 524.2 Mb with 867 scaffolds. The assembly has N50 of 2,429,002 bp, and 50% of the assembly is in the 30 longest scaffolds. Gaps in the assembly account for 240.97 bp per 100 kbp length. The final assembly has a complete BUSCO score of 86.7% and a partial BUSCO score of 5.1% to the eukaryotic database (Fig. 2a).

The mRNA-seq libraries generated 288.9 million paired-end reads. They were *de novo* assembled using Trinity (v.2.13.2) (Grabherr *et al.* 2011) producing 180,630 transcripts with N50 of 1,362 bp including 103,144 trinity genes. The BUSCO score from the nematoda_odb10 database was 63% for the transcriptome assembly and 55.2% complete for the genome assembly (Fig. 2b). This suggests that the nematode BUSCO database (nematoda_odb10) does not provide a good representation of the *M. nigrescens* genome, further highlighting the high genetic diversity among different nematode clades.

*R. culicivorax* is the only other mermithid nematode with a publicly available whole-genome assembly (Schiffer *et al.* 2013) to date. Its genome assembly has a 322 Mb size with N50 of 17,632 bp. It has 62,537 scaffolds and 37.3% complete BUSCO genes (nematoda_odb10, n = 3131). Between these 2 assemblies, the *M. nigrescens*’ genome assembly is more contiguous and complete and can be used as a better representative of mermithid nematodes.

### Genes and repeats annotation

A total of 12,313 protein-coding genes and 24,186 mRNAs including their isoforms were identified in the genome. The mean gene length is 12,224 bp, and the total gene length is 150.5 Mb. The longest gene annotated is 206,129 bp (Table 2). Functional annotation resulted in 7,496 genes annotated to the InterPro and Pfam databases and 4,623 annotated to GO terms (Supplementary Table 3). Quality of annotation was measured using Annotation Edit Distance (AED) score, and 96% of the annotated genes have an AED score of 0.5 or less, ensuring highly confident gene prediction (Supplementary Fig. 1).

**Table 2.** Genome annotation summary for *Mermis nigrescens*.

Total sequence length (bp)	524,220,005
Number of genes	12,313
Number of mRNAs (isoforms counted)	24,185
Number of exons	152,563
Number of introns	128,378
Number of CDS	24,185
Total gene length (bp)	150,457,507
Total mRNA length (bp)	298,270,450
Total exon length (bp)	30,890,614
Total intron length (bp)	267,636,592
Total CDS length (bp)	23,759,814
Longest gene (bp)	206,129
Longest mRNA (bp)	206,129
Longest exon (bp)	6,519
Longest intron (bp)	129,245
Longest CDS (bp)	19,164
Mean gene length (bp)	12,219
Mean mRNA length (bp)	12,333
Mean exon length (bp)	202
Mean intron length (bp)	2,085
Mean CDS length (bp)	982

The repeat contents in the genome amount to 440.21 Mb comprising 83.98% of the whole assembly. It includes 17.86% of retroelements, 21.97% of DNA transposons, 1.09% rolling circles, 37.18% unclassified repeatomes, and 5.23% of tandem repeats (Supplementary Table 4). Compared to other nematode species, this is a very high proportion of repeats in the genome. For example, the repeat content in *R. culicivorax* genome is 47% (Schiffer *et al.* 2013), in *T. spiralis* genome it is 19.8% (Mitreva *et al.* 2011) and in *C. elegans* genome it is 17% (*C. elegans* Sequencing Consortium 1998). At present, we do not know the significance of such a highly repetitive genome in *M. nigrescens*. However, further investigations might shed light on its biological and evolutionary significance.

## Conclusions

This study presented a high-quality genome assembly, annotation, and repeat analysis of *M. nigrescens*. The genome was assembled using the long and linked-read sequencing data. Transcriptomic data from different developmental stages of the nematode were also generated. The *M. nigrescens* genome showed very high level of repeat content compared to other nematode genomes. These new resources and information will contribute to the better understanding of the genomic architecture and biology of mermithid nematodes and their adaptation to a broad range of insect host.

## Supplementary Material

jkae023_Supplementary_Data

## Data Availability

The genome sequencing reads, RNA-seq reads, and the genome assembly for *M. nigrescens* were submitted to NCBI under the BioProject accession number: PRJNA802644. The genome assembly and the annotation can also be accessed from FigShare: https://doi.org/10.6084/m9.figshare.21761501.v1. The pipeline and the scripts used in this project are available on GitHub: https://github.com/upendrabhattarai/Nematode_Genome_Project. Supplemental material available at G3 online.
