# Supplementary material for: Genome assembly and annotation of the mermithid nematode Mermis nigrescens
Source: G3 (Bethesda). 2024 Feb 1;14(4):jkae023. doi: 10.1093/g3journal/jkae023 (PMC10989877; doi:10.1093/g3journal/jkae023)
Supplement: jkae023_Supplementary_Data [file jkae023_supplementary_data.docx]

**Supplementary materials**

**Genome assembly and annotation of the mermithid nematode *Mermis nigrescens***

Upendra R. Bhattarai^1,2^, Robert Poulin^3^, Neil J. Gemmell^1^*, Eddy Dowle^1^*

1 Department of Anatomy, University of Otago, Dunedin 9016, New Zealand

2 Department of Organismic & Evolutionary Biology, Harvard University, Cambridge, MA 02138, USA

3 Department of Zoology, University of Otago, Dunedin 9016, New Zealand

* Corresponding authors

**Keywords**: *Mermis nigrescens*, Nematode, Hybrid genome assembly, Repeatome, Genome annotation

Supplementary Table S1. Sequencing output from Oxford nanopore minion.

| Reads | Bases | Median Read Length | N50 length | Median Read Quality |
| --- | --- | --- | --- | --- |
| 530,888 | 6,935,586,657 | 11,833 | 17,849 | 13.63 |

Supplementary Table S2. Assembly statistics after each round of processing.

| Assembly steps | Assembly length | No. of scaffolds | N50 | L50 | Ns per 100 kbp | BUSCO % from Quast (eukaryota_odb10) | |
| --- | --- | --- | --- | --- | --- | --- | --- |
|  |  |  |  |  |  | Complete | Partial |
| Flye | 714,265,163 | 16,414 | 94,974 | 2,079 | 0.45 | 88.45 | 4.29 |
| Purgehaplotigs | 547,497,121 | 8,101 | 125,056 | 1,325 | 0.57 | 85.15 | 7.59 |
| Lrscaff | 739,840,003 | 5,500 | 263,548 | 890 | 2122.06 | 88.45 | 3.96 |
| LRGapcloser | 739,845,718 | 5,500 | 263,608 | 890 | 283.38 | 88.45 | 3.96 |
| Rails & Cobbler | 740,070,390 | 5,454 | 266,049 | 885 | 256.75 | 89.11 | 3.30 |
| Ragtag | 740,076,890 | 5,389 | 266,578 | 883 | 257.63 | 89.11 | 3.30 |
| ArbitR | 738,644,384 | 4,958 | 308,460 | 738 | 262.84 | 89.11 | 3.30 |
| ARKS & LINKS | 738,665,484 | 4,747 | 328,769 | 687 | 265.69 | 88.45 | 3.96 |
| Rascaf | 738,576,542 | 4,343 | 388,132 | 575 | 253.68 | 88.45 | 3.96 |
| Purgehaplotigs | 587,968,296 | 3,113 | 443,562 | 412 | 244.20 | 86.14 | 5.61 |
| Ragtag | 588,012,496 | 2,671 | 533,508 | 328 | 251.70 | 86.14 | 5.61 |
| Blobtools | 524,163,746 | 1541 | 553,974 | 285 | 228.13 | 80.86 | 5.94 |
| Ragtag | 524,231,146 | 867 | 2,428,984 | 30 | 240.95 | 80.86 | 6.27 |
| Pilon | 524,220,005 | 867 | 2,429,002 | 30 | 240.96 | 86.70 | 5.10 |

Supplementary Table S3. Functional annotation statistics from different databases.

| Database | No. of terms linked to mRNA | No. of mRNA with term | No. of gene with term |
| --- | --- | --- | --- |
| InterPro | 21,702 | 16,007 | 7,496 |
| Gene Ontology | 20,530 | 10,180 | 4,623 |
| Pfam | 21,954 | 16,007 | 7,496 |

Supplementary Table S4. Repeat content analysis in *Mermis nigresens* genome assembly.

| No. of sequences: | 867 | | | |
| --- | --- | --- | --- | --- |
| Total length (bp): | 524,220,005 | | | |
| GC level: | 36.63% | | | |
| Total repeats: | 440,214,036 bp (83.98%) | | | |
|  |  | Numbers* | Length (bp) | Percentage |
| Retroelements |  | 305,036 | 93,643,203 | 17.86% |
|  | SINEs: | 2,942 | 369,353 | 0.07% |
|  | Penelope | 25,117 | 7,072,788 | 1.35% |
|  | LINEs: | 147,865 | 40,170,331 | 7.66% |
|  | CRE/SLACS |  |  | 0.00% |
|  | L2/CR1/Rex | 85,577 | 23,760,597 | 4.53% |
|  | R1/LOA/Jockey | 80 | 54,133 | 0.01% |
|  | R2/R4/NeSL | 166 | 29,796 | 0.01% |
|  | RTE/Bov-B | 13,943 | 3,447,682 | 0.66% |
|  | L1/CIN4 | 13,163 | 2,091,210 | 0.40% |
|  | LTR elements: | 154,229 | 53,103,519 | 10.13% |
|  | BEL/Pao | 8,192 | 2,959,770 | 0.56% |
|  | Ty1/Copia | 731 | 84,377 | 0.02% |
|  | Gypsy/DIRS1 | 98,159 | 41,038,229 | 7.83% |
|  | Retroviral | 17,348 | 3,301,532 | 0.63% |
| DNA transposons |  | 503,758 | 115,186,810 | 21.97% |
|  | hobo-Activator | 105,906 | 20,614,222 | 3.93% |
|  | Tc1-IS630-Pogo | 36,016 | 7,078,658 | 1.35% |
|  | En-Spm |  |  | 0.00% |
|  | MuDR-IS905 |  |  | 0.00% |
|  | PiggyBac | 1,737 | 704,735 | 0.13% |
|  | Tourist/Harbinger | 25,116 | 5,166,803 | 0.99% |
|  | Other (Mirage, P-element, Transib) |  |  | 0.00% |
| Rolling-circles |  | 28,792 | 5,739,966 | 1.09% |
| Unclassified: |  | 777,756 | 194,902,752 | 37.18% |
| Total interspersed repeats: |  |  | 403,732,765 | 77.02% |
| Small RNA: |  | 15,557 | 2,652,405 | 0.51% |
| Satellites: |  | 2,895 | 658,174 | 0.13% |
| Tandem repeats: |  | 165,073 | 27,430,726 | 5.23% |
| Simple repeats: |  |  |  | 0.00% |
| Low complexity: |  |  |  | 0.00% |
| * Most repeats fragmented by insertions or deletions have been counted as one element. | | | | |


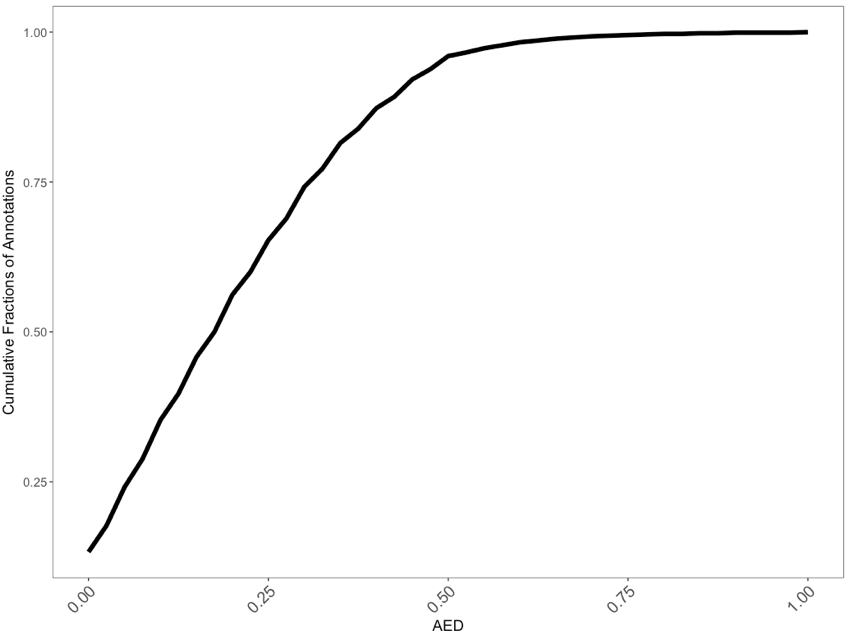


Supplementary Figure S1: Annotation quality with AED scores. The y-axis shows the cumulative fractions of annotations and the x-axis their AED scores.
